# β3-Adrenergic receptor antagonism improves cardiac and vascular functions but did not modulate survival in a murine resuscitated septic shock model

**DOI:** 10.1186/s40635-024-00705-9

**Published:** 2024-12-18

**Authors:** Eugénie Hagimont, Marc-Damien Lourenco-Rodrigues, Benjamin-Glenn Chousterman, Frances Yen-Potin, Manon Durand, Antoine Kimmoun

**Affiliations:** 1 Inserm U1116, DCAC, Université de Lorraine, Nancy, France; 2 APHP, CHU Lariboisière, Département d’anesthésiologie et Réanimation, Inserm, MASCOT Paris, Université Paris Cité, Paris, France; 3 CHRU de Nancy, Service de Médecine Intensive et Réanimation Brabois, INSERM U116, F-CRIN-INI-CRCT, Université de Lorraine, Vandœuvre-Lès-Nancy, France

**Keywords:** Septic shock, Receptor, Beta-3, Adrenergic, Vascular reactivity

## Abstract

**Background:**

Recent findings suggest that β3-adrenergic receptors (β3-AR) could play a role in the hemodynamic regulation, but their function in septic shock remains unclear. This study investigates the modulation of β3-AR in an experimental murine model of resuscitated septic shock on in vivo hemodynamic, ex vivo vasoreactivity, inflammation and survival.

**Method:**

Wild-type mice were used, undergoing cecal ligation and puncture (CLP) to induce septic shock, with SHAM as controls. Mice were treated with β3-AR agonist or antagonist three hours post-CLP, followed by resuscitation with fluids and antibiotics. Hemodynamic parameters were measured at 18 h following the surgery, and the expression of β-ARs in heart and aorta was assessed via immunostaining and western blot. Vascular reactivity was studied using myography, and inflammatory markers were analyzed through PCR and western blots. A 5-day survival study was conducted, documenting clinical severity scores and survival rates.

**Results:**

β3-AR was expressed in both endothelial and myocardial cells in healthy and septic mice. During septic shock model, β3-AR density on endothelial cells increased post-CLP, while β1- and β2-AR decreased or remained constant. β3-AR antagonist treatment improved hemodynamic parameters, increasing mean arterial pressure and cardiac index, unlike the agonist. Vascular reactivity to phenylephrine was enhanced in aortic rings from both β3-AR agonist and antagonist-treated mice. However, no significant differences in inducible NO synthase expression were observed among treated groups. Despite improved hemodynamic parameters with β3-AR antagonist treatment, survival rates in treated groups remained similar to CLP group.

**Conclusions:**

In an experimental murine model of resuscitated septic shock, β3-AR is resistant to desensitization and its inhibition improves cardiac and vascular function without affecting the short-term survival.

**Supplementary Information:**

The online version contains supplementary material available at 10.1186/s40635-024-00705-9.

## Take-home message

1. Experimental septic shock enhanced β3-AR expression on endothelial cells.
2. β3-AR antagonism improves cardiac and vascular functions but does not impact 5-day survival rates.

## Background

Septic shock is defined as a life-threatening organ dysfunction triggered by a dysregulated host response to infection that can lead to death [1]. Despite advances in the management of septic shock, the global incidence, estimated from studies in high-income countries, remains high, representing at least 19.4 million cases with potentially 5.3 million deaths annually [2]. In 2020, the World Health Organization prioritized the improvement of sepsis and septic shock management as one of its goals. The pathophysiology of septic shock, while extensively studied, remains a complex and partially understood syndrome. In specific, it is known that high levels of catecholamines are associated with impaired sympathetic nervous system function, characterizing sympathetic dysautonomia during septic shock [3–5]. This phenomenon leads to the overstimulation of β-adrenergic receptors (β-ARs), resulting in impaired cardiac function, altered vasoreactivity, and immunoparalysis [6]. In this context, we previously demonstrated that β1-AR blockade was associated with sepsis-induced immunosuppression through modulation of regulatory T-cell inhibitory function [7]. Although initially raising great hopes, β1-AR modulation later proved to be inconclusive in clinical trials and even potentially dangerous [8, 9]. While the involvement of β1- and β2-ARs in septic shock is now well‐described, the β3-AR remains the least characterized. This receptor, initially described in brown adipose tissue and involved in thermogenesis in newborns, has been detected in several human tissues, including the myocardium, retina, kidney and bladder [10]. In the myocardium, β3-AR stimulation is known to exert a negative inotropic effect, presumably through a nitric oxide (NO) pathway [11, 12]. While β3-AR has not been easily evidenced in vessels, nebivolol, a selective β1-AR blocker and β3-AR agonist, is already prescribed for hypertensive and/or heart failure patients. Its vasodilating effects appear to be NO-mediated through β3-AR stimulation [13]. Moreover, unlike other β-ARs, β3-AR is not susceptible to desensitization [14]. Considering all these biological effects, β3-AR should also be involved in the pathophysiology of septic shock. Surprisingly, it has been poorly studied, with very few experimental studies conducted [15–18]. Thus, our objective is to comprehensively examine the impact of modulating β3-AR on cardiac and vascular functions in an experimental resuscitated septic shock model in mice.

## Methods

### Animals

Wild-type (WT, C57BL/6J, DBA/2, 129Sv) mice were used for this study after regeneration from sperm cryopreserved in straws with C57Bl/6J females. Mice were housed in central animal facility of the Faculty of Medicine in Nancy. The French Animal Care Committee, in accordance with European regulations, approved all protocols (n°APAFIS#34184). Details on the origin of WT and knockout β1-AR^−/−^ mice are described in the supplementary methods.

### Septic shock model

#### CLP surgery

Septic shock was induced by cecal ligation and puncture (CLP) in both male and female mice aged 12–16 weeks, weighing 20–40 g. In non-treated groups (SHAM), mice only underwent a laparotomy with exposure of the caecum. The detailed procedure, performed according to the literature, is presented in Supplemental Data Material—Methods [19].

#### Resuscitation

Three hours after the surgery, the mice received an intraperitoneal fluid bolus (5 ml/100 g, 0.9% NaCl) containing a broad-spectrum antibiotic therapy (0.01 mg/g, imipenem 500 mg, cilastatine 500 mg, Tienam, MSD, Kenilworth). All mice received the same amount of fluid per unit of body weight.

#### β3-AR agonist and antagonist treatments

β3-AR agonist (CL316243, a phenylethanolamine: C5976, Merck KGaA, Darmstadt, Germany) and β3-AR antagonist (SR59230A, an aryloxypropanolaminotetraline: S8688, Merck KGaA, Darmstadt, Germany) were administered once at 0.001 mg/g jointly with the fluid bolus three hours after the surgery. The doses of agonists and antagonists were selected based on literature from studies using a murine model of sepsis [20].

#### Experimental design

The mice were acclimatized for 5 days in the experimental area. Following surgery, they were randomly assigned to one of four groups: SHAM, CLP, CLP+ agonist, and CLP+ antagonist. Three hours after waking up from surgery, mice were resuscitated and received the interventions (agonist or antagonist). Then, mice were placed in a specific post-operative area, at room temperature, and they were left to rest for 15 h. Eighteen hours post‐CLP, mice underwent measurements and experimentations. Of note, the number of mice used and reasons for failed measurements and for exclusion are provided in Supplemental Data Material—Table S1.

### Measurements and experimental protocols

#### Echocardiography

Transthoracic echocardiography was performed using the VEVO3100 (FUJIFILM VisualSonics, Toronto, Canada). A full description is provided in Supplemental Data Material—Methods. Mice were placed on a heating pad and the analysis began when mice temperature reached 36 °C. Mean arterial pressure (MAP) was measured through a catheter inserted into the left carotid artery with a blood pressure transducer (ACQ7700, DSI Harvard Biosciences, Holliston, USA).

#### Vascular reactivity procedure

Mice were killed, the thoracic aorta and mesenteric artery were collected and their vasoreactivity was studied by ex vivo myography. The procedure is detailed in Supplemental Data Material—Methods.

#### Immunostaining

The aortic tissue was embedded in O.C.T. (Lamb/OCT, ThermoFisher Scientific, Waltham, USA) and flash frozen in liquid nitrogen. Six-micrometer cryosections were performed using a cryostat (Leica Microsystems, Wetzlar, Germany). After fixation with 4% paraformaldehyde, the histological sections of aorta were labeled with the primary antibodies (Supplemental Data Material—Table S2) of interest, and then detected with fluorescent secondary antibodies.

Quantifications were carried out using ImageJ software (National Institutes of Health, USA). The images were not analyzed in a blinded manner. The area of interest was manually traced and measured in square micrometers. The integrated density was determined for each traced area. Background fluorescence intensity was measured. Corrected Tissue Fluorescence (CTF) was calculated using the following formula:

$$ {\text{CTF}} = {\text{Integrated}}\;{\text{density}}{-}\left( {{\text{Area}}\;{\text{of}}\;{\text{selected}}\;{\text{tissue}} \times {\text{Average}}\;{\text{fluorescence}}\;{\text{of}}\;{\text{background}}\;{\text{readings}}} \right). $$

The calculation of the proportions from the CTF values was carried out in this way per animal for each group:

$$ \frac{{{\text{Sum}}\;{\text{of}}\;{\text{CTF}}\;{\text{values}}\;{\text{of}}\;{\text{each }}\upbeta {\text{ - AR in each mouse}}}}{{{\text{Sum of CTF values of each }}\upbeta {\text{ - AR in all mice}}}} \times 100 $$

#### Polymerase chain reaction

Total RNA extraction was carried out with the RNA Plus mini-Kit (74104, Qiagen NV, Venlo, The Netherlands) according to the manufacturer’s instructions. The extracted RNAs were reverse transcribed into cDNA using the kit manufacturer’s instructions and in BioRad iCycler iQTM (1708891, BioRad, Hercules, USA). The sequences of interest, described in Supplemental Data Material—Table S3), were hybridized to the probes and amplified with the BioRad CFX Connect-Real-Time System (Hercules, USA).

#### Western blot

Proteins from the heart, thoracic aorta and mesenteric arteries were extracted using a lysis buffer containing 25 mM bicine buffer (pH 7.6) with phosphatase inhibitor and complete protease inhibitor reagents (04719956001, Roche, Basel, Switzerland). The protein concentration was determined using the Bradford reagent. After separation on SDS-PAGE gels, proteins were transferred to nitrocellulose membranes, which were incubated overnight at 4 °C with primary antibodies (Supplemental Data Material–Table S4) after a blocking step. A chemiluminescent signal was produced using ECL^+^ solution (170-5060, BioRad Hercules, USA) and detected using a Fusion FX imager (Vilber, Marne-la-Vallée, France). Relative densitometry was performed using Image MultiGauge 3.0 software (Fujifilm, Tokyo, Japan). Protein normalization was conducted using the total protein method with Ponceau Red staining.

#### Survival study

Survival was studied in CLP, CLP+ agonist and antagonist groups. The animal census was conducted twice a day for 5 days. Administration of agonist or antagonist began three hours post-CLP and was reinjected intraperitoneally daily (for both agents, half-life = 16 h and dose = 0.001 mg/g) with buprenorphine (dose = 0.0002 mg/g) in a bolus of 200 µL (0.7 ml/100 g). The clinical severity score and weight of each mouse were measured daily. This score includes several items such as appearance, provoked behavior, unprovoked behavior and hydration status (Supplemental Data Material—Table S5). This clinical score ranging from 0 to 15 was employed to evaluate the daily condition of the mice. The endpoint to decide euthanasia for the 5-day survival study was defined as a clinical score > 10 (Supplemental data material—Table S5), in combined with a weight loss greater than 10%.

#### Sample size

The sample size was determined, in accordance with the Animal Ethics Committee, on our previous study employing the same murine CLP model [7]. Consequently, eight animals per group were deemed necessary.

#### Statistics

Data in the tables are presented as medians with 25th and 75th percentiles, or with min–max when *n* < 5. Comparisons between the SHAM and CLP groups were presented using Hodges–Lehmann estimates with 95% confidence intervals and no further tests were performed for vasoreactivity, western blot and RT-PCR analyses. Comparisons among the CLP, CLP+ agonist, and CLP+ antagonist groups in tables and figures were performed using the Kruskal–Wallis test. Post-hoc pairwise comparisons were conducted using Dunn’s test. Kaplan–Meier curves were plotted for the survival study, and the log-rank test was used for comparisons. Analyses of weight changes and clinical severity scores were performed using a linear mixed model with random effects for each mouse and time point. The assumption of normality for the residuals was assessed using both the quantile–quantile (Q–Q) plot and the W statistic from the Shapiro–Wilk test. No statistical tests were performed when *n* < 5. A *p* value < 0.05 was considered statistically significant. Statistical analyses were performed using GraphPad Prism version 8.0.2 (GraphPad Software, San Diego, CA) and R version 4.3.3 (2024-02-29).

## Results

### Endothelial β3-AR expression in healthy mice

**Fig. 1:**
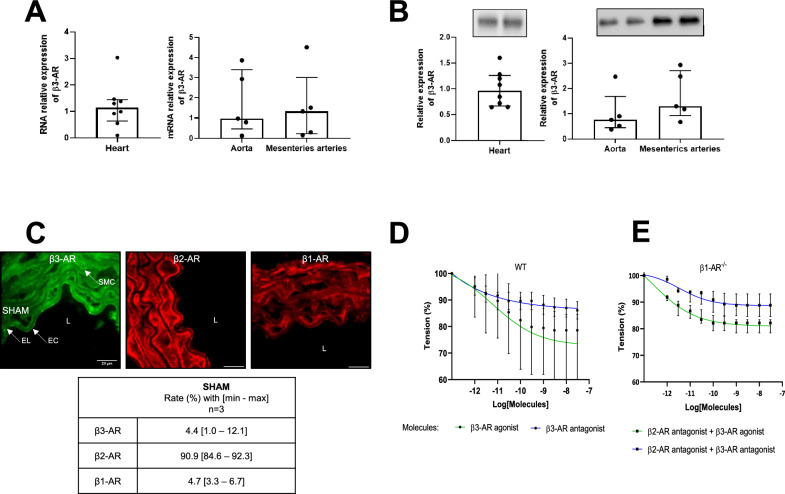
Expression of β3-AR in heart, aorta and mesenteric arteries of healthy mice. **A** Relative mRNA expression of β3-AR in healthy heart, aorta and mesenteric arteries using RT-PCR (heart *n* = 8 and vessels *n* = 5). **B** Relative expression of β3-AR protein in the heart, aorta and mesenteric arteries using western blot analysis (heart *n* = 8 and vessels *n* = 5). **C** Representative fluorescence images of β3-AR (green), β2-AR (red) and β1-AR (red) in the aorta. Magnification ×100, scale bar 20 µm. EC: Endothelial Cell, EL: Elastic Lamina, L: Lumen, SMC: Smooth Muscle Cell. For all images, the arrows localize the endothelium. The quantified area used for the analyses is indicated by the EC arrow. The table presents the proportion of β-AR subtypes by immunostaining. Data are expressed with median, minimum and maximum (*n* = 3, no statistic). **D** Relaxation of aortic rings from healthy mice (in percent) as a function of increasing concentrations of agonist and antagonist expressed as log [M, mole/L] after pre-contraction with phenylephrine (*n* = 4, no statistics). **E** Relaxation of aortic rings from β1-AR^−/−^ mice with β2-AR blocked using an ICI118551 pre-treatment (*n* = 4, no statistics). Data in panels A, B D and E are expressed with median [25th;75th]

We located and quantified the expression of the β3-AR compared to the other β-ARs and assessed its functional activity. Figure 1A and B shows the expression of β3-AR at both transcriptional and protein levels in heart, aortic and mesenteric arteries. Using immunostaining technique, as shown in Fig. 1C, β-ARs were detected on the endothelium, where β2-AR labeling density was higher than those of β1-AR and β3-AR (β2-AR 90.9% [84.6–92.3], β1-AR 4.7% [3.3–6.7], β3-AR 4.4% [1.0–12.1]). Finally, using a myograph bench, increasing doses of the agonist induced vasorelaxation in control aortic rings from wild-type mice, while those of antagonist did not (Fig. 1D). The specificity of these responses persisted even after pharmacological blockade of β2-AR and genetic knockout of β1-AR (Fig. 1E).

### Impact of β3-AR modulation on hemodynamic in a resuscitated septic shock model

In a resuscitated septic shock model, the impact of agonist and antagonist was assessed on macrohemodynamic indices such as MAP and cardiac function. Supplemental Data Material—Table S7 summarizes the hemodynamic of mice. Eighteen hours after inducing the septic shock with CLP, mice exhibited a slight reduction in HR (SHAM median [25th; 75th percentiles]: 469 [423; 484] vs. CLP 410 [339; 443]/min, Hodges–Lehmann estimates: − 48 95%CI [− 120; 25]) and a decrease in MAP (SHAM 96.1 [85.8; 100.3] vs. CLP 61.3 [60.3; 66] mmHg, Hodges-Lehmann estimate: − 32.9 95%CI [− 39.5; − 22.2]).

In CLP mice, the addition of an antagonist resulted in an overall improvement in hemodynamic parameters compared to mice receiving resuscitation alone (Table 1). Specifically, MAP was significantly increased in the CLP+ antagonist group compared to the CLP group (CLP 61.3 [60.3; 66] vs. CLP+ antagonist 80.8 [67.7; 87.9] mmHg, *p* = 0.007). In addition, the cardiac index (CI) and cardiac power index CPI were also increased by the antagonist, with no significant difference in HR values (CI: CLP 0.40 [0.32; 0.43] vs. CLP+ 0.59 [0.43; 0.65] ml/min/g, *p* = 0.018; CPI: CLP 0.056 [0.040; 0.062] vs. CLP+ antagonist 0.100 [0.075; 0.120] W/g, *p* = 0.006). Conversely, the agonist did not induce any macrohemodynamic improvements in MAP, CI or CPI.

**Table 1:** Hemodynamic parameters in CLP mice without or with agonist or antagonist treatments

Parameters	CLP *N* = 7 Median [25th; 75th]	CLP + agonist *n* = 8 Median [25th; 75th]	CLP + antagonist *n* = 8 Median [25th; 75th]	Global *p* value	Post hoc tests
Weight (g)	25.3 [24 ; 27.4]	26.6 [22.6 ; 29.7]	24 [22 ; 26.1]	0.546	CLP vs. CLP + agonist: > 0.999 CLP vs. CLP + antagonist: 0.747
Heart rate (bpm)	410 [339 ; 443]	430 [378 ; 465]	439 [400 ; 465]	0.559	CLP vs. CLP + agonist: 0.628 CLP vs. CLP + antagonist: 0.774
Mean arterial pressure (mmHg)	61.3 [60.3 ; 66] (*n* = 6)	60.6 [60.5 ; 72] (*n* = 7)	80.8 [67.7 ; 87.9]	0.007	CLP vs. CLP + agonist: 0.528 CLP vs. CLP + antagonist: 0.007
Stroke volume (µl)	24 [17.1 ; 28.1]	28.5 [23.3 ; 36.3]	36.1 [26.4 ; 38.6]	0.042	CLP vs. CLP + agonist: 0.218 CLP vs. CLP + antagonist: 0.025
Cardiac index (ml/min/g)	0.40 [0.32 ; 0.43]	0.49 [0.40 ; 0.59]	0.59 [0.43 ; 0.65]	0.029	CLP vs. CLP + agonist: 0.134 CLP vs. CLP + antagonist: 0.018
Cardiac power index (W/g)	0.056 [0.040 ; 0.062] (*n* = 6)	0.078 [0.65 ; 0.098] (*n* = 7)	0.100 [0.075 ; 0.120]	0.006	CLP vs. CLP + agonist: 0.114 CLP vs. CLP + antagonist: 0.006

CLP vs. CLP + agonist or CLP + antagonist tested with a Kruskal-Wallis test and a post hoc Dunn’s test as appropriate

### Impact of β3-AR modulation on β-AR proportions

**Fig. 2:**
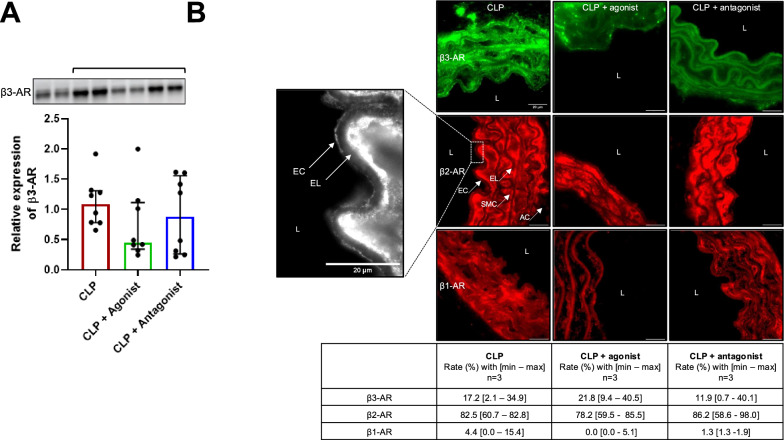
Expression of the β-AR subtypes in the heart and aorta of septic shock mice without or with agonist or antagonist treatments. **A** Relative expression of β3-AR protein in the heart using western blot analysis (heart *n* = 8). The bracket indicates the corresponding blot. Data are expressed with median [25th;75th]. **B** Representative fluorescence images of β3-AR (green), β2-AR (red) and β1-AR (red) in the aorta. Magnification ×100, bar scale 20 µm. EC: Endothelial Cell, EL: Elastic Lamina, L: Lumen, SMC: Smooth Muscle Cell. For all images, the arrows localize the endothelium. The quantified area used for the analyses is indicated by the EC arrow. The table represents the proportion of β-AR subtypes. Data are expressed with median, minimum and maximum (*n* = 3, no statistic)

No apparent differences in the protein expression of heart β3-AR was found between the SHAM and CLP groups, nor between the CLP and CLP+ treatment groups (Supplemental Data Material—Figure S2, panel A and Fig. 2, panel A). Aorta β-ARs proportions were quantified after immunostaining and revealed that septic shock altered the proportions of β-ARs on the aortic endothelium. β3-AR levels increased, from 4.4% [1.0–12.1] in the SHAM group to 17.2% [2.1–34.9] in the CLP group, those of β2-AR and β1-AR remained relatively constant (Supplemental Data Material—Figure S2, panels B and C). Figure 2B shows that, compared to the CLP group, β1-AR proportions in treated groups showed a trend toward zero. The proportions of β2- and β3-AR appeared unchanged in the treated groups compared to the CLP group.

### Impact of β3-AR modulation on vasoreactivity

**Fig. 3:**
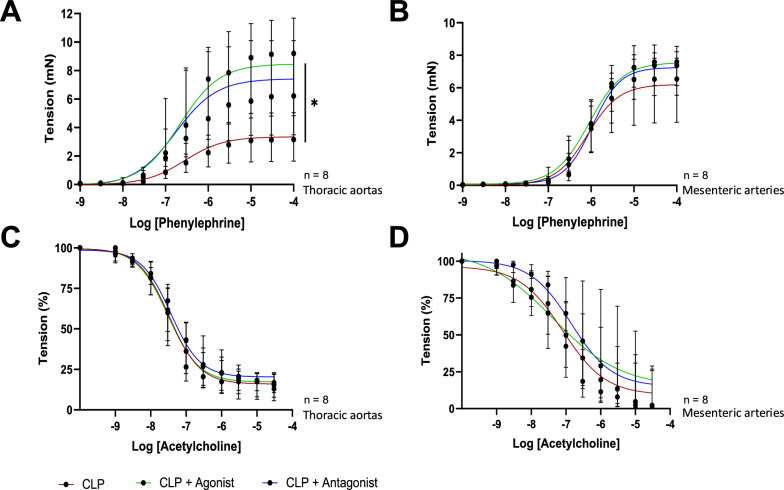
Ex vivo vascular reactivity to phenylephrine and concentration–response curves to acetylcholine using thoracic aortic and mesenteric artery rings of septic shock mice without or with agonist or antagonist treatments. **A**, **B** Contraction of the vessel (in mN) as a function of increasing concentrations of phenylephrine (Phe) expressed as log of Phe [M, mole/L]. **C**, **D** Relaxation of the vessel (in percent) as a function of increasing concentrations of acetylcholine (Ach) expressed as log of Ach [M, mole/L]. Data are expressed with median [25th;75th] (*n* = 8 per group)

We investigated the contraction and relaxation capacities of both aortic and mesenteric rings in response to increasing doses of phenylephrine and acetylcholine. Supplemental Data Material—Figure S3 shows the contraction and relaxation capacities in SHAM and CLP groups. Figure 3A shows that aortic rings in the CLP+ agonist and CLP+ antagonist groups displayed enhanced contractility compared to rings from the CLP untreated group (*p* = 0.017 and *p* = 0.080 respectively). Conversely, no difference was found between groups regarding mesenteric arteries contractile capacities (*p* = 0.224). The maximum relaxation capacity remained unmodified in both aortic and mesenteric rings when septic shock mice were treated with agonist or antagonist compared to CLP (*p* = 0.555 an *p* = 0.150 respectively, Fig. 3C and D).

### Impact of β3-AR modulation on inflammatory and metabolic parameters

**Fig. 4:**
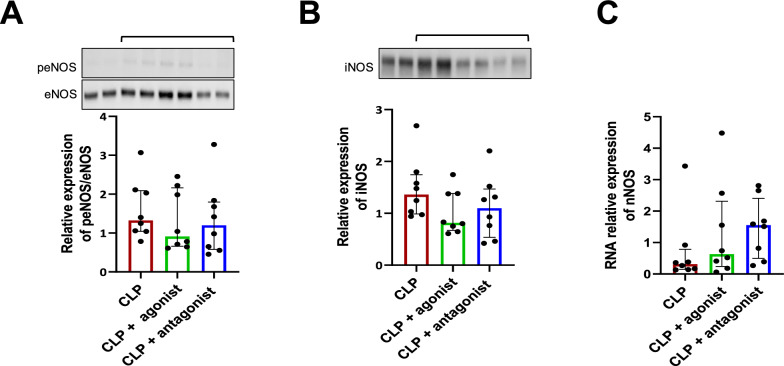
Analysis of NOS isoforms in the heart of Septic shock mice without or with agonist or antagonist treatments. **A** and **B** Relative protein expressions of peNOS/eNOS and iNOS in the heart using western blot. The bracket indicates the corresponding blot. **C** RNA relative expression of nNOS in the heart using RT-PCR. Data are expressed in median [25th;75th] (*n* = 8 per group)

The effects of agonist or antagonist treatments were assessed on heart tissue. Transcriptional expression of VCAM-1 and nNOS, protein expression of iNOS, NFκB and phosphorylated endothelial Nitric Oxide Synthase (peNOS)/eNOS were presented for SHAM and CLP in Supplemental Data Material—Figures S4 and S5. The administration of agonist or antagonist did not affect the expression of peNOS/eNOS compared to the CLP group (*p* = 0.505, Fig. 4A). The expression of iNOS protein was not altered in the treated groups compared to the CLP group (*p* = 0.178, Fig. 4B). The CLP+ antagonist group seemed to exhibit higher nNOS transcription levels compared to the CLP group (*p* = 0.095, Fig. 4C). In addition, the transcriptional and/or protein analysis of E-selectin, VCAM-1, ICAM-1 and NFκB did not reveal any significant effect of the agonist or antagonist treatments (Supplemental Data Material—Figure S5, panels B, D, F and G). No difference among groups was found in lung wet/dry weight ratio or in VE-cadherin expression (*p* = 0.084, Supplemental Data Material—Figure S6). The influence of β3-AR modulation on energetic metabolisms was also studied. Triglyceride levels appear decreased in the CLP group compared to the SHAM group (SHAM: 33.2 [18.3; 46.5] vs. CLP: 17.3 [9.5; 24] mg/dl, Hodges–Lehmann estimate: − 16.2 95%CI [− 32.9; − 0.6], Supplemental Data Material—Table S7), and administration of an agonist tended to restore these levels toward normal values (CLP: 17.3 [9.5; 24] vs CLP+ agonist: 25.1 [15.6; 37.5] mg/dl, *p* = 0.176, Supplemental Data Material—Table S8).

### Impact of β3-AR modulation on survival in a resuscitated septic shock model

**Fig. 5:**
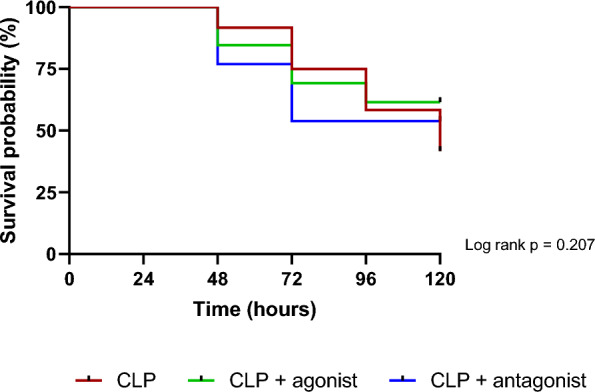
Five-day survival curves in septic shock mice with or without agonist or antagonist treatments. CLP: *n* = 12, CLP+ agonist: *n* = 13 and CLP+ antagonist: *n* = 13. Data were analyzed with the Log-rank test

*C*linical score severity was used as a health indicator in all groups of mice during the 5-day survival study. In the SHAM group, the severity score remained at 0 throughout the study period. In contrast, all CLP groups exhibited an increase in the severity score to 4 [3–5] (Supplemental Data Material—Figure S7, panel A) with no global difference after treatments compared to CLP alone. The weights were also unaltered over time and groups (Supplemental Data Material—Figure S7, panel B). The survival rate was documented in CLP, CLP+ agonist or antagonist mice (Fig. 5). Median survival time in CLP group was 120 h. Survival at 120 h was unchanged among groups (log rank *p* = 0.207).

## Discussion

In this study, we first demonstrate that β3-AR are expressed on both endothelial and myocardial cells in mice. Second, in an experimental resuscitated septic shock model, β3-AR density is increased on endothelial cells. Third, modulation of β3-AR by an antagonist is associated with improved hemodynamic status, although this does not translate into any survival benefit.

### Expression and modulation of β3-AR in myocardial and endothelial cells

Before investigating the effects of β3-AR modulation on the cardiovascular function in septic shock mice, we first determined protein and RNA expression levels of these ARs in endothelial cells of both healthy and septic mice. Several studies, including ours, have reported the expression of β3-AR in myocardial tissues, and in cultured cells [21–24]. In contrast, very few studies reported the distribution of β3-ARs levels in endothelial cells from peripheral arteries [25, 26]. In our study, we confirmed the presence of β3-AR in endothelial cells from aortic arteries both directly by immunostaining and indirectly by functional vasoreactivity tests.

### Septic shock increases the proportion of β3-AR on endothelial cells

We hypothesized that unlike β1- or β2-ARs, β3-AR, is not prone to desensitization in critical states. While septic shock appears to reduce the expression of β2- and β1-ARs on aortic endothelial cells, the proportion of β3-AR increases or remains constant. Previous research has shown that β3-AR is resistant to agonist-mediated desensitization because it lacks a phosphorylation site for β-ARK [14]. This unique feature may be crucial in septic shock, where excessive β-AR stimulation causes desensitization, leading to reduced vascular responsiveness to catecholamines and increased ICU mortality [27, 28].

### Modulation of β3-AR improves the hemodynamic

In the CLP+ antagonist group, the hemodynamic was improved. With an unaltered HR, changes in CI and MAP must be attributed to an elevated stroke volume likely due to either improved venous return or enhanced inotropic activity. It is difficult to reasonably determine from echocardiographic-derived hemodynamic parameters whether the antagonist exerts its effect on the heart or on the vessels. However, Moniotte et al. found that β3-ARs are upregulated in the myocardium of septic patients and that a β3-AR agonist worsened the amplitude of contractile shortening in LPS-incubated myocytes [16]. Thus, during septic shock, the upregulation of β3-AR in response to high catecholamine levels may lead to a negative inotropic effect [11, 29]. In the present study, β3-AR antagonist might therefore have restored the cardiac function [30].

### Modulation of β3-AR improves vasoreactivity

To explain the increased SV, we also hypothesized that improved venous return could be attributed either to enhanced arterial and/or venous vasoconstriction or to reduced vascular leakage [31]. In our study, vascular responsiveness to phenylephrine was enhanced in aortic rings from septic shock mice treated with either β3-AR agonist or antagonist, an intriguing result given both agents led to similar improvements. By contrast, Evans blue test and lung wet/dry ratio, which assess the vascular leakage, were unaltered by the interventions. While β3-ARs are usually associated with vasorelaxation, their role in regulating vascular contractility during septic shock remains under-investigated. However, in a rodent heart failure model, administration of a β3-AR antagonist improved vascular responsiveness to norepinephrine [32]. The role of β3-AR modulation in improving the vasoreactivity is complex, with both β3-AR agonist and antagonist potentially enhancing vasoreactivity through different mechanisms.

### Modulation of the β3-AR does not alter NO production

As expected, we observed an increase in iNOS protein expression in CLP mice. However, modulation of the β3-AR did not affect the protein or RNA expression levels of the three NOS isoforms. Reconciling our results with the existing literature is challenging, as studies present conflicting findings regarding the underlying mechanisms of β3-AR modulation [10, 17, 20]. Our untested hypothesis is that the improvement in vasoconstriction is linked to a balanced activity of NOS isoforms and a reduction in oxidative stress such as demonstrated in a model of pulmonary hypertension treated with a β3-AR agonist [33].

### Modulation of the β3-AR did not translate in survival benefit

Although we clearly demonstrated an improvement in the hemodynamic pattern with the administration of an antagonist, the survival study did not show any benefit from the modulation of β3-AR. This result could be seen as unexpected, as an improvement in hemodynamics should have enhanced survival. However, similar conflicting results have been reported by others. In an LPS model, some researchers reported that early administration of an antagonist was associated with a dramatic increase in survival [20]. Conversely, another group concluded, also in an LPS inflammatory model, that β3-AR does not play a significant role in regulating LPS-mediated mortality [34].

### Limitations

First, while we used a CLP murine model known for closely mimicking human septic shock, it has its limitations. At 18 h post-CLP, the mice consistently exhibited bradycardia, which is uncommon in humans. Key factors contributing to this include (1) low body mass, which favors hypothermia and partially causes the observed bradycardia; (2) earlier sympathetic overactivation, leading to dysautonomia [35] and (3) reduced parasympathetic response compared to humans, further decreasing HR [36]. Second, although the murine adrenergic system is considered an excellent surrogate for the human system, it has certain specificities. For example, rodent and human β3-ARs differ in their expression between white and brown adipocytes [37]. Moreover, CL316243 stimulates the production of cAMP more effectively in rodents than in humans. Nevertheless, it is widely used due to its high selectivity, being 129 times more selective for β3-ARs than for β1-ARs [38, 39]. Third, a major limitation of this model is the ability to precisely control both the onset of shock and the initiation of treatments, which contrasts with the unpredictable timing observed in human settings. Fourth, in the survival study, the antibiotic treatment was administered only once, while fluids and other treatments were renewed daily. Finally, the absence of observed biological effects from the interventions in our study could partly be attributed to (1) a moderate severity of the CLP; (2) the use of non-comorbid young mice [40, 41]; (3) the inclusion of both male and female animals, potentially reducing the statistical power of the study. Indeed, estrogens are known to exert protective effects in sepsis [42, 43]. Moreover, we initially planned a high-grade CLP with a 75% mortality rate at 2 days but only a 50% mortality rate was observed at 5 days. Increasing the sample size and a prolonging the follow-up period might have altered the results.

## Conclusion

In an experimental murine model of resuscitated septic shock, β3-AR does not appear to undergo desensitization processes. Inhibition of β3-AR is associated with improved cardiac and vascular functions; however, no effect was found on 5-day survival. Further experimental research is essential to understand the underlying mechanisms before considering any clinical application.

## Supplementary Information

Supplementary Material 1.

## Data Availability

All the material published in this study could be found on https://osf.io/a98xy.

## Abbreviations

β-AR: β-Adrenergic receptor
β-ARK: β-Adrenergic receptor kinase
cAMP: Cyclic adenosine monophosphate
CI: Cardiac Index
CLP: Cecal ligation and puncture
CPI: Cardiac Power Index
CTF: Corrected tissue fluorescence
e-i-nNOS: Endothelial–inducible–neuronal NO synthase
HR: Heart Rate
I-VCAM-1: Intercellular–vascular adhesion molecule 1
ICU: Intensive care unit
IQR: Interquartile
LPS: Lipopolysaccharides
MAP: Mean arterial pressure
NFκB: Nuclear Factor kappa-light-chain-enhancer of activated B cells
NO: Nitric oxide
PAF: Paraformaldehyde
PCR: Polymerase chain reaction
peNOS: Phosphorylated endothelial NO synthase
RNA: Ribonucleic acid
WT: Wild-type
