# Supplementary material for: β3-Adrenergic receptor antagonism improves cardiac and vascular functions but did not modulate survival in a murine resuscitated septic shock model
Source: Intensive Care Med Exp. 2024 Dec 18;12:118. doi: 10.1186/s40635-024-00705-9 (PMC11655894; doi:10.1186/s40635-024-00705-9)
Supplement: Supplementary file 1 — Supplementary Material 1. [file 40635_2024_705_MOESM1_ESM.docx]

**β3-Adrenergic receptor antagonism improves cardiac and vascular functions but did not modulate survival in a murine resuscitated septic shock model**

***Supplemental digital content***

**SUMMARY**

[**Animals** 3](#_Toc182658924)

[**Septic shock model** 3](#_Toc182658925)

[**Echocardiography** 4](#_Toc182658926)

[**Arterial pressure measurement** 4](#_Toc182658927)

[**Vascular reactivity procedure** 5](#_Toc182658928)

[**Immunostaining** 7](#_Toc182658929)

[**Western Blot** 12](#_Toc182658930)

[**Blood count** 13](#_Toc182658931)

[**Metabolism dosage** 13](#_Toc182658932)

[**Lung wet/dry weight ratio** 14](#_Toc182658933)

[**Reference** 14](#_Toc182658934)

[Supplemental Digital Content – Tables 15](#_Toc182658935)

[**Table S1: Summary of mouse distribution by procedure.** 15](#_Toc182658936)

[**Table S2: Primary antibodies in immunostaining.** 16](#_Toc182658937)

[**Table S3: Sequences of mouse primers.** 17](#_Toc182658938)

[**Table S4: Western blot primary antibodies.** 18](#_Toc182658939)

[**Table S5: Clinical Score.** 19](#_Toc182658940)

[**Table S6: Preparation of standard range and samples.** 20](#_Toc182658941)

[**Table S7: Hemodynamic, blood count and metabolic parameters in SHAM and septic shock mice** 21](#_Toc182658942)

[**Table S8: Hemodynamic, blood count and metabolic parameters in septic shock mice without or with agonist or antagonist treatments.** 24](#_Toc182658943)

[Supplemental Digital Content – Figures 27](#_Toc182658944)

[**Figure S1: Endothelial location of β3-AR in healthy murine aorta.** 27](#_Toc182658945)

[**Figure S2: Quantification of β3-AR in the heart and in the thoracic aorta of SHAM and septic shock mice.** 28](#_Toc182658946)

[**Figure S3: Ex-vivo vascular reactivity to phenylephrine and concentration–response curves to acetylcholine using thoracic aortic and mesenteric resistance artery rings in SHAM and septic shock mice.** 30](#_Toc182658947)

[**Figure S4: Analysis of NOS isoforms in heart tissue in SHAM and septic shock mice.** 31](#_Toc182658948)

[**Figure S5: Analysis of inflammatory biomarkers in heart tissue in SHAM and septic shock mice without or with β3-AR modulation.** 32](#_Toc182658949)

[**Figure S6: Evaluation of the vascular permeability in septic shock mice.** 34](#_Toc182658950)

[**Figure S7: Variation in clinical score and weight of septic shock mice over five days.** 35](#_Toc182658951)

**Supplemental Digital Content – Methods**

## **Animals**

β1-AR^-/-^ mice were generated by the department of molecular pharmacology of Roche Bioscience. Briefly, the targeting vector of the endogenous β1-AR genomic locus was constructed using sequence that had been cloned from a 129sv mouse genomic library. The gene of the β1-AR was disrupted in the targeting vector by placing Escherichia coli lacZ gene encoding b-galactosidase and neomycin resistance gene. Embryonic stem (ES) cells were electroporated and selected for neomycin resistance. ES where homologous recombination occurred were injected into C57Bl/6 blastocysts to obtain germ line chimeras. Chimeric males were then mated to DBA/2 female mice to screen for germ line transmission of the ES cell DNA. Then heterozygote mice were intercrossed to generate β1-AR^+/+^ (WT) and β1-AR^-/-^ mice. After obtaining β1-AR^-/-^ mice in the laboratory, heterozygotes were generated by backcross with C57BL/6J mice. Mice were genotyped and two strains, β1-AR^-/-^ and these littermates β1AR^+/+^ (WT) were used in this study.

## **Septic shock model**

Before surgery, the mice received a subcutaneous injection of an analgesic, buprenorphine, at a dose of 0.0002 mg/g (Buprécare®, Axience, Pantin, France). Anesthesia was induced using 3% isoflurane (Isorane®, Axience, Pantin, France) and maintained at 2% throughout the procedure. The body temperature was maintained at 38°C using a heating plate. The mice’s abdomen was shaved, and the skin was disinfected to ensure adherence to good surgical practices. A midline laparotomy was performed involving an incision through the skin and peritoneum to expose the cecum. The latter was ligated at the ileocecal valve obstructing 75% of the appendix. A puncture was made using an 18G needle and feces were extruded to induce the peritonitis. The cecum was then reintroduced into the peritoneal cavity and the different tissue layers were sutured. The SHAM mice underwent a laparotomy without ligation and puncture of the cecum. All mice received a subcutaneous bolus injection of physiological saline (1 ml, 0.9% NaCl) after the surgery. Three hours after the surgery, animals were resuscitated with an intraperitoneal fluid bolus injection (5 ml/100g, 0.9% NaCl) containing a broad-spectrum antibiotic therapy (0.01 mg/g of Imipenem 500mg, Cilastatine 500mg, Tienam, MSD, Kenilworth).

## **Echocardiography**

A transthoracic echocardiogram was performed 18 hours after surgery with a VEVO3100 and a probe MX550D. Anesthesia was induced with 2.5% and maintained with 1.5% isoflurane. Mice were placed on a heating pad and the analyzes began when mice temperature (monitored using a rectal probe) reached 36°C. The thorax part of was shaved, and the skin was cleaned before applying a pre-warmed ultrasound gel.

Stroke volume (SV) and cardiac index (CO) were calculated with the following formulas: SV(µl) = VTI(cm) x π x AD(cm)^2^/4; CI (ml/min/g) = HR (/min) x SV(µl)/weigth (g). The cardiac power index (CPI) was calculated with the following formula: CPI (W/g) = MAP(mmHg) x CI(ml/min/g) / 451. All measurements were averaged over three consecutive cardiac cycles.

## **Arterial pressure measurement**

*Procedure.* After performing the echocardiography, anesthesia was maintained with 1.5% isoflurane. A skin incision was made in the middle of the anterior cervical region. The muscles were dissected, and the left carotid artery was isolated and separated from the vagus nerve. The distal end of the carotid artery was ligated with a suture while the proximal end was clamped using an arterial clamp. The incision of the carotid artery was made using a micro-scissor. A catheter (PE-50, Braintree Scientific®, Braintree, Chicago, USA) pre-filled with a heparinized solution (50 IU/ml of 0.9% NaCl) was inserted into the carotid artery towards the proximal end and held in place using wires suture. The pressure transducer (ACQ7700, DSI, Harvard Biosciences, Holliston, USA) was connected to the catheter and the arterial clamp was removed. Arterial pressure was acquired and analyzed with Ponemah software (Data Science International, New Brighton, USA) for five to ten minutes until a stable signal was obtained. At the end of the procedure, 500 µl of blood was collected through the catheter in a tube containing citrate (100µl citrate for 500µl of blood, 04.1922, Sarstedt, Nümbrecht, Germany).

## **Vascular reactivity procedure**

*General procedure.* A pressure myograph system was used for all vasoreactivity procedures (Multi myograph system 610M, Danish Myo Technology, Denmark). Before the start of the experiments, a calibration was carried out. Aorta and intestinal tissues were gently removed. With the aid of a microscope, first-order mesenteric arteries were isolated in iced-cooled physiological saline solution (PSS) (mM: NaCl 130, KCl 3.7, MgSO_4_ 7H_2_O 1.2, NaHCO_3_ 14.9, CaCl_2_ 2 H_2_O 1.6, HEPES 5, KH_2_PO_4_ 1.2, D-Glucose 11). Once the aorta and mesenteric arteries were cleared of adipose tissue, 2 mm rings were cut. The aorta rings were mounted directly on the hooks. For the mesenteric artery rings, two 40 µm tungsten wires were passed through the lumen and then were mounted one on the upper jaw connected to a force sensor and the other on the lower jaw. Each ring was bathed in a 37°C warmed solution of 8 ml PSS, renewed every 20 minutes. Aeration to maintain proper oxygen levels with a gas mixture of 95% oxygen and 5% carbon dioxide was applied to the organ bath chamber. Then the rings were pre-stretched to a level that approximates physiological conditions. The tension was set between 75 and 80 mmHg for all vessels and a maximum passive force at 7.5 mN for the aortas and 4.8 mN for the mesenteric arteries. After a rest and stabilization period of 45 minutes, the maximum contraction capacity of the vessels was tested with two steps: 1/ depolarization with 8 ml at 2,9% of KCl, 2/ depolarization with 8 mL of KCl 2.9% added to 16 µl of phenylephrine at 10^-3^ M, a α1-AR agonist. After two PSS washes and a 20-minute washout period, phenylephrine concentration-response curves were induced from 10^-9^ M to 3 × 10^-4^ M in the organ bath chambers. The endothelium-dependent capacity for vessel relaxation of phenylephrine-precontracted vessels was studied using concentration-response curves to acetylcholine from 10^-9^ M to 3.10^-4^ M.

For each day of experimentation, two mice were tested. For each mouse, two rings of large (conduit) aorta vessels and two rings of small (resistance) mesenteric vessels were assessed and averaged. *Ex vivo* experiments didn’t exceed three to four hours. The technique was performed following current recommendations [1].

*Selectivity of β3-AR agonist and antagonist on healthy aorta rings.* Relaxation responses to β3-AR agonist and antagonist were tested on aorta rings from four healthy WT and four knockout β1-AR^-/-^ mice. The concentration-response curves of vessels pre-contracted to phenylephrine were recorded with increasing doses of β3-AR agonist, CL316243 and β3-AR antagonist, SR59230A ranging from 10^-11^ M to 3.10^-7^ M.

In four additional β1-AR^-/-^ mice, relaxation responses to the β3-AR agonist and antagonist, as described above, was also tested in the presence of a β2-AR antagonist, ICI118551 (L127, Merck KGaA, Darmstadt, Germany), added in the chambers at 10^-7^ M. This final experiment tested the selectivity of the β3-AR agonist and antagonist with both β1- and β2-ARs blocked.

## **Immunostaining**

*Procedure.* Tissue sections were fixed with 4% PAF for 15 minutes. After washing with 1X TBS-T, the sections were then permeabilized with 0.1% Triton for ten minutes. After washing with 1X TBS-T, the sections were treated with 3% hydrogen peroxide for ten minutes. After washing with 1X TBS-T for 5 minutes, saturation of tissue sections was performed with 1X TBS-T containing 5% BSA for 30 minutes. The primary antibodies diluted in 1X TBS-T containing 1% BSA were placed incubate for one night at 4°C (**Supplemental Digital Content – Table S2**). After three washes with 1X TBS-T, the fluorescent secondary antibodies (anti-mouse 488 nm A21202, anti-rabbit 555 nm A21429, ThermoFisher Scientific, Waltham, USA) were incubated for one hour at room temperature. After three washes with 1X TBS-T, nuclei were stained with 4′,6-diamidino-2-phenylindole (DAPI) for 15 minutes. Sections were washed three times with 1X TBS-T before mounting with Fluoromount^-GTM^. Images (magnification ×40 and ×100) were collected using a fluorescence microscope Nikon ECLIPSE Ci (Tokyo, Japan) with identical capture settings for each stain to confirm its removal.

*Epifluorescence microscope observations.* Each section was observed at x100 magnification using oil with the Nikon ECLIPSE Ci. For each animal, three fields at x100 magnification were taken to carry out quantifications. The same parameters were kept for each cut: exposure 700 ms.

*Quantification.* For each field, three areas at the elastic blades were selected for background noise. For each field, three areas of the endothelium were manually delineated to quantify only endothelial protein expression. The intensity and integrated density values of each area were used to determine the expression level of our protein.

Corrected tissue fluorescence (CTF) was calculated using this formula: Integrated density – (selected tissue area x average background fluorescence intensity).

Negative values, ​​corresponding to background noise exceeding marker expression, were set at zero.

*Proportions.* For each animal and each β-AR, CTF averages were performed in each condition. The proportions were calculated as follows:

$$\frac{\text{Sum of CTF values ​​of each β-AR in each mouse}}{\text{ Sum of CTF values ​​of β-AR in all mice}}\text{ x100}$$

Data are expressed as median with minimum and maximum values in percent. No statistical analysis was performed.

**Polymerase chain reaction**

*Procedure.* Preparation of RNA samples: Heart, mesentery, and thoracic aorta were removed and placed in PBS on ice. Adipose tissues were removed from the mesenteric and aortic vessels. The samples were ground with liquid nitrogen to obtain a powder. The powder was dissolved and homogenized in 1 ml of TRIzol Reagent extraction buffer. To separate the different phases of the extraction, 200 µl of chloroform was added. The samples were kept at room temperature between 15 minutes and one hour for complete destruction of cell membranes. The samples were centrifuged for 15 minutes at 10 000 g. The upper phase of each sample was collected in a new tube and frozen at - 80°C.

*Extraction and dosages of total RNA.* RNAs were collected, extracted and purified following the instructions of the manufacturer's extraction protocol (74104, Qiagen NV, Venlo, The Netherlands). The RNAs were heated to 70°C to obtain single-stranded RNAs for 5 minutes then immediately cooled on ice before being assayed and stored at -80°C.

The RNA concentration was determined by measuring the absorbance at 260 nm (NanoDrop, ThermoFisher Scientific, Waltham, USA).

Extraction and dosages of mRNA: For aorta and mesenteric arteries: After total RNA extraction, between 724 and 1000 ng of RNA were collected per sample. Samples were heated at 65°C for 2 minutes to linearize the RNA strands, then immediately stored on ice.

Preparation of DynabeadsTM magnetic beads: In a tube, 1 mg of DynabeadsTM magnetic beads (61006, ThermoFisher Scientific, Waltham, USA) was resuspended then placed on the magnet for 30 seconds allowing adhesion of the DynabeadsTM magnetic beads to the wall of the tube. The supernatant was collected and discarded. The DynabeadsTM magnetic beads were resuspended in 100 µl of binding buffer to equilibrate the beads. The supernatant was collected and discarded in the same way, thanks to the adhesion of the DynabeadsTM magnetic beads to the wall of the tube placed on the magnet. A volume of binding buffer equal to the volume of RNA was added to the DynabeadsTM magnetic beads to allow optimal hybridization conditions.

*mRNA isolation.* The total RNA sample was added to the DynabeadsTM magnetic beads. Hybridization was carried out at room temperature with stirring for five minutes. The mRNA-DynabeadsTM magnetic bead complexes were washed twice with 200 µL of washing buffer. The RNAs were eluted with 16 μl of 10 mM Tris-HCl buffer at pH 7.5. The sample was placed at 70°C for two minutes and then immediately placed on ice. The eluted mRNA was collected in a new tube and then assayed.

*Retro-transcription.* The complementary DNAs (cDNAs) were generated from the RNAs obtained previously. For each sample, a mixture is made, composed of RNA (500 ng), the Iscript Synthesis kit mix and made up to 20 µL with nuclease-free H2O (1708891, BioRad, Hercules, USA). After five minutes at 25°C of enzyme activation, the cDNAs were synthesized for 20 minutes at 46°C followed by an enzyme inactivation step for one minute at 95°C in the BioRad iCycler iQTM (Hercules, USA).

*Polymerase chain reaction.* The 20 µL of cDNA are diluted one-fifth with nuclease-free water to obtain a final volume of 100 µL. A mixture consisting of 12.5 µL of Sybr Green (1708887, BioRad, Hercules, USA) 2X, 0.75 µL of sense and anti-sense primers (**Supplemental Digital Content – Table S3**) at tenµM and six µL nuclease-free water is made and added to each well containing five µL of diluted cDNA. Each sample is deposited in duplicate. The PCR cycle used includes three steps after three minutes of pre-heating the cDNA to 95°C:

Step 1: Denaturation of the cDNA is carried out at 95°C for ten seconds to obtain a single-stranded cDNA.

Step 2: Hybridization of the primers to the complementary single-stranded cDNA at 62°C for ten seconds.

Step 3: Extension of the primers by DNA polymerase at 72°C for 30 seconds.

This cycle is repeated 40 times and followed by a melting step at 95°C for ten seconds and at 65°C for five seconds then stopped at 95°C for five seconds in the BioRad CFX Connect-Real-Time System (Hercules, USA).

For the analysis of the results, RPS29 was used as the reference gene.

## **Western Blot**

*Acrylamide gel.* A 10% acrylamide gel was made according to the manufacturer's instructions (161-0183, BioRad, Hercules, USA). The 35 µg proteins were prepared in a volume of 95 µL total containing 11.3 µL of β-mercaptoethanol diluted tenth in Laemmli Blue (161-0747, BioRad, Hercules, USA) and supplemented with distilled water. The proteins, as well as a two-color weight marker of 10 to 250 kDa (161-0374, BioRad, Hercules, USA) were deposited in the wells. Each sample was deposited in duplicate.

*Migration.* Protein migration was carried out using a 10X TRIS-glycine-SDS (TGS) migration buffer (161-0772, BioRad, Hercules, USA) diluted 10 times, first at 60V for 15 minutes then at 120V between 30 minutes and one hour.

*Transfer.* The proteins are then transferred to a 0.2 μm nitrocellulose membrane (1704159, BioRad, Hercules, USA) using the Trans-Blot® TurboTM System (BioRad, Hercules, USA) and the membrane was stained with Ponceau red (A40000279, ThermoFisher Scientific, Waltham, USA. The membrane was washed twice with distilled water and once with 1X Tris Buffered Saline (TBS) prepared with TBS10X (Tris Base 10X, NaCl 1X, H2O, pH7.5-7.6) and 0.001% Tween 20. The membranes were cut between 50 and 100 kDa to incubate with different antibodies.

*Marking.* The membranes were saturated with five mL of 1X TBS-T containing 5% milk proteins for one hour with shaking, washed with 1X TBS-T and incubated overnight at 4°C with the primary antibodies (**Supplemental Digital Content – Table S4**). After three washes, the secondary antibodies diluted to the thousandth anti-rabbit Immunoglobulin G (IgG) (NA931V, GE Healthcare, Chicago, USA) and anti-mouse IgG (NA934V, GE Healthcare, Chicago, USA) were incubated for one hour. Three washes were carried out before revealing using the Clarity^TM^ kit according to the manufacturer's instructions (170-5060, BioRad, Hercules, USA) on the device. The bands were revealed using the Fusion FX imager (Vilber, Marne-la-Vallée, France).

To reuse the membranes, dehybridization was carried out for two minutes using the Stripping Buffer (21059, ThermoFisher Scientific, Waltham, USA). After three washes with 1X TBS-T, the membranes are saturated, then incubated with another antibody. The dehybridization of the antibodies was checked, and repeated if necessary.

Quantifications were carried out on total proteins, or by the ratio of total proteins to phosphorylated proteins, using MultiGauge 3.0 software (Fujifilm, Tokyo, Japan).

## **Blood count**

 In 7 to 8 mice from each group, complete blood counts were measured (Horiba, Kyoto, Japan). The blood was centrifuged for ten minutes at 8 000 g at 18°C to recover the plasma.

## **Metabolism dosage**

Glucose, triglycerides, cholesterol determination: Each assay was performed according to the Libios colorimetric assay kit manufacturer's instructions (Vindry Sur Turdine, France). For each assay, a standard range and the plasma samples were deposited in duplicate in a micro-titration plate according to the following volumes: **Supplemental Digital Content – Table S6**. The plate was left on the shaker for ten minutes to allow the enzymatic reaction to take place. Absorbance was measured at 510 nm in a plate reader. Concentrations in milligrams per deciliter were obtained by following the formula: $\frac{\text{Absorbance sample}}{\text{Absorbance standard}}$  x 100.

## **Lung wet/dry weight ratio**

Eighteen hours after surgery, the five lung lobes (left, middle, right anterior, right posterior and accessory) were removed and weighed. They were placed in a dry oven at 50°C for 24 hours then reweighed. A ratio of wet weight to dry weight of the lungs was carried out to evaluate the volume of fluid having passed through the vascular walls to infiltrate the lung tissues.

## **Reference**

1. Wenceslau CF, McCarthy CG, Earley S, et al (2021) Guidelines for the measurement of vascular function and structure in isolated arteries and veins. Am J Physiol Heart Circ Physiol 321:H77–H111. https://doi.org/10.1152/ajpheart.01021.2020

# **Supplemental Digital Content – Tables**

## **Table S1: Summary of mouse distribution by procedure.**

| Experiment | Set | N experiment | N excluded* | Measurements performed in n= | Reason of exclusion |
| --- | --- | --- | --- | --- | --- |
| Echocardiograph | 1 | 36 | 5 | 31 | Mice 1,2,3: death before echography  Mice 4, 5: values > 3 SD  (echocardiography) |
| Blood pressure | 1 |  |  | 29 |  |
| Blood count | 1 |  |  | 30 |  |
| Myography | 2 | 32 | 0 | 32 |  |
| Survival study | 3 | 44 | 0 | 44 |  |

^*Excluded means that the mice were excluded from all analysis^

## **Table S2: Primary antibodies in immunostaining.**

| Protein | Reference | Supplier | Dilution |
| --- | --- | --- | --- |
| β3-AR | sc515763 | SCBT, Dallas, USA | 1/200 |
| β2-AR | SAB4500577 | Merck KGaA, Darmstadt, Germany |  |
| β1-AR | 12271S | CST, Danvers, USA |  |
| vWF | A0082 | Agilent, Santa Clara, USA |  |

## **Table S3: Sequences of mouse primers.**

| Mouse primers | Sequences | Efficacity | Dilution |
| --- | --- | --- | --- |
| β3-AR | GAC TAC AGA CCA TAA CCA ACG TG  CCT GGT GGC ATT ACG AGG A | 95.3 | 1/5 |
| eNOS | ATC ACC TAC CGA CAC CCT CAG  CGG CTC TGT AAC TTC CT TGG | 90.2 |  |
| VCAM-1 | CCC GTC ATT GAG GAT ATT GG  GGT CAT TGT CAC AGC ACC AC | 100.3 |  |
| ICAM-1 | TTC ACA CTG AAT GCC AGC TC  GTC TGC TGA GAC CCC TCT TG | 101.6 |  |
| E-Selectin | TCA ACT TGA GTG CAC ATC TCA GG  GAT TGA AGG CTT TGG CAG CT | 88.2 |  |
| VE-Cadherin | CAG GCC CTA ACT TTC CCC AG  CAC AGT GGG GTC ATC TGC AT | 91.9 |  |
| RPS29 | ACG GTC TGA TCC GCA AAT AC  CAT GAT CGG TTC CAC TTG GT | 91.9 |  |
| nNOS | GTG GCC ATC GTG TCC TAC CAT AC  GTT TCG AGG CAG GTG GAA GCT A | 83.5 | 1/2 |

## **Table S4: Western blot primary antibodies.**

| Protein | Reference | Supplier | Dilution |
| --- | --- | --- | --- |
| peNOS, phosphorylated Ser1177 | 9570S | Saint Quentin Yvelines, France | 1/1000 |
| eNOS | 32027S | Saint Quentin Yvelines, France |  |
| β3-AR | sc515763 | SCBT, Dallas, USA |  |
| pNFκB phosphorylated Ser536 | MA5-15160 | ThermoFisher Scientific, Waltham, USA |  |
| NFκB | 510500 | ThermoFisher Scientific, Waltham, USA |  |
| iNOS | sc7271 | SCBT, Dallas, USA |  |

## **Table S5: Clinical Score.**

| Clinical score | |
| --- | --- |
| Appearence | Normal (0) |
|  | Lack of grooming (1) |
|  | Ruffled hairs (2) |
|  | Huddled (3) |
|  | Half-closed eyes (4) |
| Unprovoked behavior | Normal (0) |
|  | Minor changes (1) |
|  | Less mobile, isolated (2) |
|  | Restless or very still (3) |
| Provoked behavior | Responsive and alert (0) |
|  | Not responding or alerting (3) |
| Hydration status | Normal (0) |
|  | Dehydrated (5) |

## **Table S6: Preparation of standard range and samples.**

| Well | Volume |
| --- | --- |
| Blank | 0 µL H_2_O + 200 µL reagent |
| Standard 2 | 2 µL standard + 200 µL reagent |
| Standard 4 | 4 µL standard + 200 µL reagent |
| Standard 10 | 10 µL standard + 200 µL reagent |
| Samples | 2 µL plasma + 200 µL reagent |

## **Table S7: Hemodynamic, blood count and metabolic parameters in SHAM and septic shock mice**

| **Hemodynamic parameters** | **SHAM**  **n = 8**  **Median [25^th^ ;75 ^th^]** | **CLP**  **n = 7**  **Median [25^th^;75 ^th^]** | Hodges-Lehmann estimate **with 95%CI** |
| --- | --- | --- | --- |
| Weight (g) | 25.3 [22.4 ; 29.3] | 25.3 [24 ; 27.4] | -0.2 [-4.3; 3.7] |
| *Weight variation (g) | -1.5 [-2.2 ; -1.2] | -0.4 [-1.5 ; 1.4] | 1.4 [0.3; 2.6] |
| Clinical score | 0 [0 ; 0] | 5 [4 ; 8] | 5 [4; 8] |
| Heart rate (bpm) | 469 [423 ; 484] | 410 [339 ; 443] | -48 [-120; 25] |
| Respiratory rate (rpm) | 93 [78 ; 122} | 42 [30 ; 52] | -53 [-83; 4] |
| Mean arterial pressure (mmHg) | 96.1 [85.8 ; 100.3] | 61.3 [60.3 ; 66]  (n = 6) | -32.9 [-39.5; -22.2] |
| Stroke volume (µl) | 32.1 [28.6 ; 36] | 24 [17.1 ; 28.1] | -9.9 [-17.3; -2.6] |
| Cardiac output (ml/min) | 14.4 [12.5 ; 16.9] | 9.5 [7.6 ; 11.9] | -5.1 [-9.4; -2.1] |
| Cardiac index (ml/min/g) | 0.52 [0.45 ; 0.67] | 0.40 [0.32 ; 0.43] | -0.14 [-0.31; -0.05] |
| Cardiac power index (W/g) | 0.111 [0.085 ; 0.155] | 0.056 [0.040 ; 0.062]  (n = 6) | -0.056 [-0.107; -0.029] |
| **Blood count parameters** |  |  |  |
| Leukocytes (10^3^/mm^3^) | 4.2 [2.6 ; 6.1] | 0.8 [0.4 ; 1.5] | -3.5 [-5.5; -1.5] |
| Lymphocytes (%) | 89 [80.3 ; 91.7] | 79.3 [64.4 ; 92.1] | -11 [-22.3; 4.1] |
| Monocytes (%) | 8.1 [5.8 ; 12.4] | 14.7 {6.7 ; 25.8] | 5.1 [-2.9; 16;1] |
| Granulocytes (%) | 3.5 [1.8 ; 6.4] | 8.3 [1.2 ; 10.3] | 3.1 {-2; 7] |
| Platelets (10^3^/mm^3^) | 710 [662 ; 807] | 465 [345 ; 636] | -252 [-379; -74] |
| **Metabolic parameters** |  |  |  |
| Glucose (mg/dl) | 68.8 [33.6 ; 128.7] | 58 [42.9 ; 113.7] | -14.5 [-79.5; 43.9] |
| Triglycerides (mg/dl) | 33.2 [18.3 ; 46.5] | 17.3 [9.5 ; 24] | -16.2 [-32.9; -0.6] |
| Cholesterol (mg/dl) | 69.3 [44.4 ; 84.8] | 58.4 [31.1 ; 82.5] | -10.1 [-38.1; 25.7] |

^*Weight variation: difference between weight before and 18H after the surgery.^

## **Table S8: Hemodynamic, blood count and metabolic parameters in septic shock mice without or with agonist or antagonist treatments.**

| **Hemodynamic parameters** | **CLP**  **n = 7**  **Median [25^th^;75 ^th^]** | **CLP + agonist**  **n = 8**  **Median [25^th^;75 ^th^]** | **CLP + antagonist**  **n = 8**  **Median [25^th^;75 ^th^]** | **Global p value** | **Post hoc test** |
| --- | --- | --- | --- | --- | --- |
| Clinical score | 5 [4 ; 8] | 7 [6 ; 9] | 4 [3 ; 5] | 0.013 | CLP. vs CLP + agonist: 0.345  CLP. vs CLP + antagonist: 0.275 |
| Weight variation (g) | -0.4 [-1.5 ; 1.4] | 0.8 [0.5 ; 1.3] | -1 [-1.4 ; 1.3] | 0.0798 | CLP. vs CLP + agonist: 0.253  CLP. vs CLP + antagonist: 0.999 |
| Cardiac output (ml/min) | 9.5 [7.6 ; 11.9] | 12.5 [9.5 ; 16.3] | 14.1 [11.4 ; 16.9] | 0.037 | CLP. vs CLP + agonist: 0.136  CLP. vs CLP + antagonist: 0.025 |
| Respiratory rate (rpm) | 42 [30 ; 52] | 65 [39.3 ; 86.5] | 89 [76.8 ; 112] | 0.093 | CLP. vs CLP + agonist: 0.641  CLP. vs CLP + antagonist: 0.060 |
| **Blood count parameters** |  |  |  |  |  |
| Leukocytes (10^3^/mm^3^) | 0.8 [0.4 ; 1.5] | 1.2 [0.6 ; 1.9] | 0.7 [0.5 ; 1] | 0.344 | CLP. vs CLP + agonist: > 0.999  CLP. vs CLP + antagonist: 0.816 |
| Lymphocytes (%) | 79.3 [64.4 ; 92.1] | 70.2 [60.2 ; 76.7] | 68.8 [63.4 ; 79.9] | 0.385 | CLP. vs CLP + agonist: 0.354  CLP. vs CLP + antagonist: 0.607 |
| Monocytes (%) | 14.7 {6.7 ; 25.8] | 21.3 [16 ; 23.2] | 21.2 [14.5 ; 30.6] | 0.493 | CLP. vs CLP + agonist: 0.669  CLP. vs CLP + antagonist: > 0.515 |
| Granulocytes (%) | 8.3 [1.2 ; 10.3] | 8.4 [4 ; 14] | 8.1 [3.9 ; 10.6] | 0.807 | CLP. vs CLP + agonist: > 0.999  CLP. vs CLP + antagonist: > 0.999 |
| Platelets (10^3^/mm^3^) | 465 [345 ; 636] | 444 [278 ; 609] | 614 [382 ; 624] | 0.591 | CLP. vs CLP + agonist: 0.908  CLP. vs CLP + antagonist: > 0.999 |
| **Metabolic parameters** |  |  |  |  |  |
| Glucose (mg/dl) | 58 [42.9 ; 113.7] | 22.1 [9.1 ; 31.3] | 40.6 [19.1 ; 54.3] | 0.010 | CLP. vs CLP + agonist: 0.008  CLP. vs CLP + antagonist: 0.404 |
| Triglycerides (mg/dl) | 17.3 [9.5 ; 24] | 25.1 [15.6 ; 37.5] | 12.8 [10.6 ; 24.5] | 0.170 | CLP. vs CLP + agonist: 0.176  CLP. vs CLP + antagonist: > 0.999 |
| Cholesterol (mg/dl) | 58.4 [31.1 ; 82.5] | 47.1 [34.4 ; 56.2] | 51.4 [22.6 ; 90.3] | 0.601 | CLP. vs CLP + agonist: 0.603  CLP. vs CLP + antagonist: > 0.999 |

^*Weight variation: difference between weight before and 18H after the surge^

# **Supplemental Digital Content – Figures**

## **Figure S1: Endothelial location of β3-AR in healthy murine aorta.**

(A) Immunostaining of endothelial β3-AR (green). (B) Immunostaining of von Willebrand factor (vWF, red), a specific endothelial marker indicating the integrity of the endothelium. (C) After removing the endothelium, absence of endothelial β3-AR immunostaining. (D) After removing the endothelium, poorly vWF immunostaining. L: Lumina. Magnification x40 (n = 3). For all figures, arrows localize the endothelium.

**
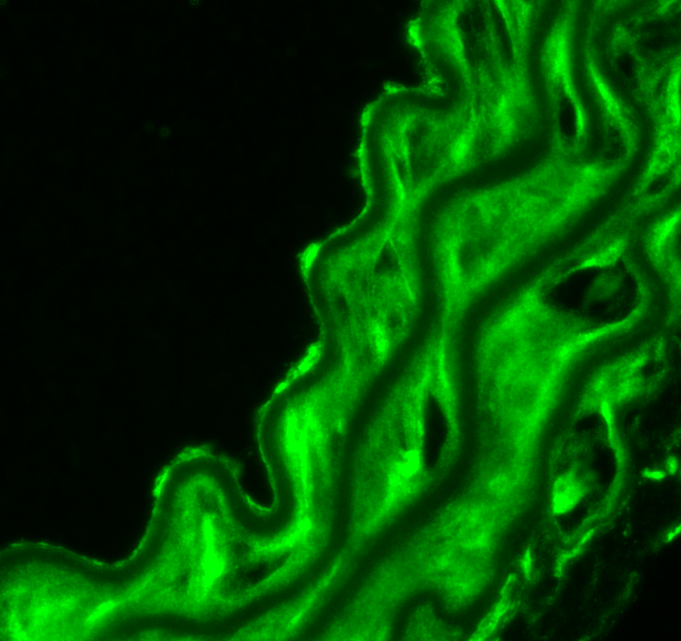

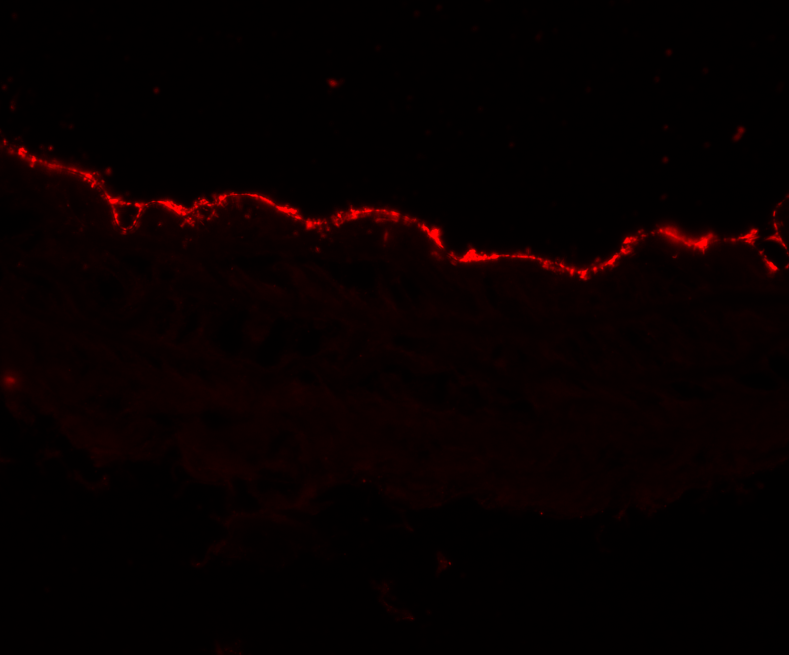
A B**

L

L

**
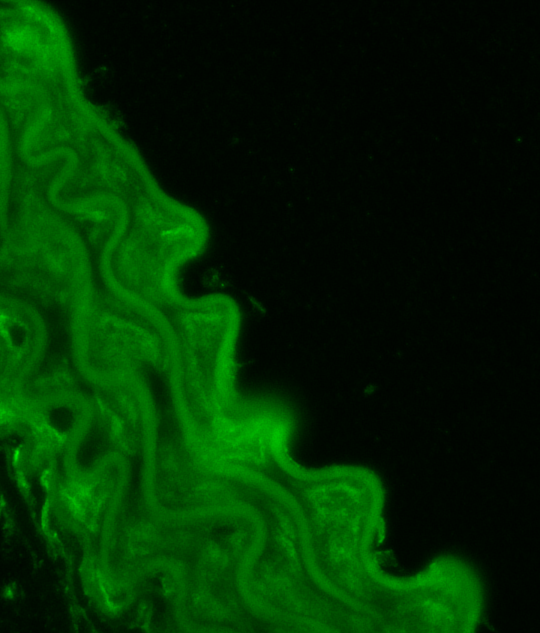

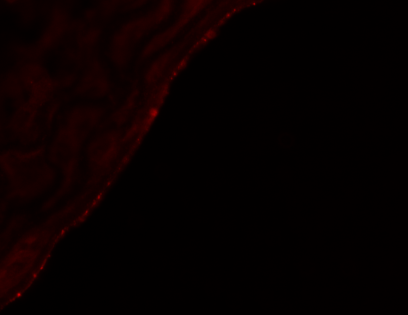
C D**

L

L

## **Figure S2: Quantification of β3-AR in the heart and in the thoracic aorta of SHAM and septic shock mice.**

(A) Relative β3-AR protein expression using Western blot (n = 8 per group). Data are expressed with median [25^th^; 75 ^th^]. The bracket indicates the corresponding blot. (B) Proportions of the three β-AR subtypes: β1-AR, β2-AR, and β3-AR in the thoracic aorta of SHAM and CLP mice, as determined by immunostaining (n = 3 per group). Data are expressed as median with minimum and maximum values (in percent, no statistical analysis). (C) Images representing the negative controls of β-AR markers for each experimental condition by immunostaining. L: Lumina. Magnification x100. For all figures, arrows localize the endothelium.

**
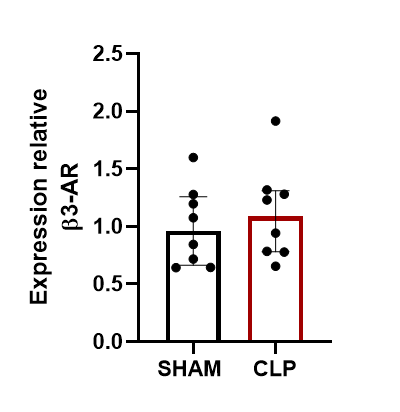
**
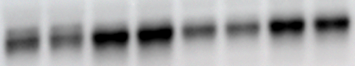
**A**

β3-AR

**B**

|  | SHAM | Rate (%) with [min – max]  n=3 | CLP | Rate (%) with [min – max]  n=3 |
| --- | --- | --- | --- | --- |
| β3-AR | 4.4 [1.0 – 12.1] | | 17.2 [2.1 – 34.9] | |
| β2-AR | 90.9 [84.6 – 92.3] | | 82.5 [60.7 – 82.8] | |
| β1-AR | 4.7 [3.3 – 6.7] | | 4.4 [0.0 – 15.4] | |

**C**

CLP + antagonist

CLP + agonist

CLP

SHAM

Negative β1, β2-ARs

Negative β3-AR

L


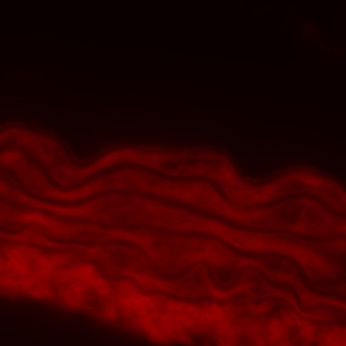

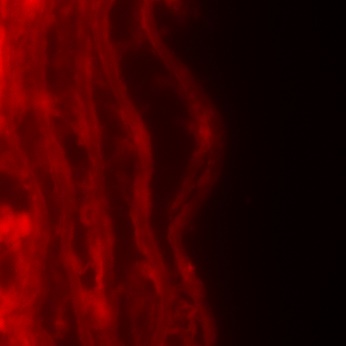

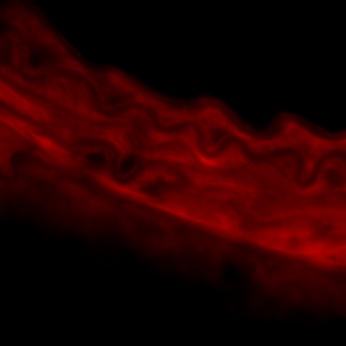

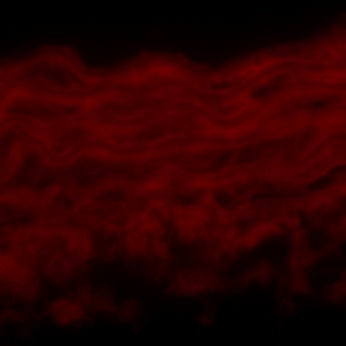

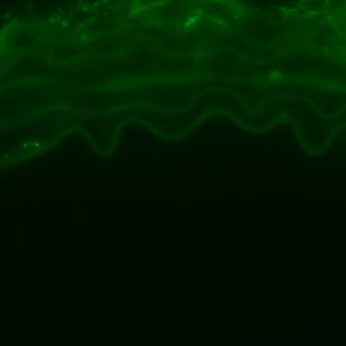

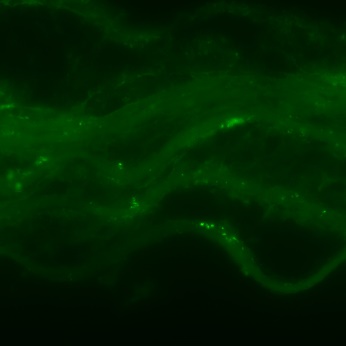

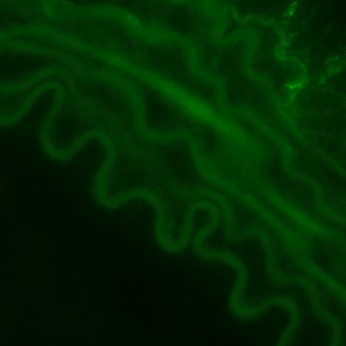

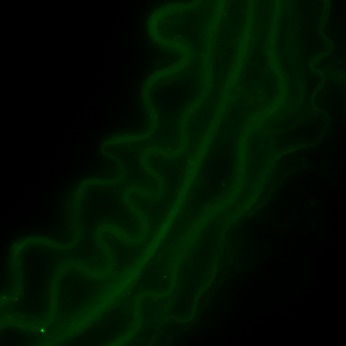


L

L

L

L

L

L

L

**Figure S3: Ex-vivo vascular reactivity to phenylephrine and concentration–response curves to acetylcholine using thoracic aortic and mesenteric resistance artery rings in SHAM and septic shock mice.**

(A, B) Contraction of the vessel (in mN) as a function of increasing concentrations of phenylephrine (Phe) expressed as log of Phe [M, mole/L]. (C, D) Relaxation of the vessel (in percent) as a function of increasing concentrations of acetylcholine (Ach) expressed as log of Ach [M, mole/L]. Data are expressed with median [25^th^; 75 ^th^] (n = 8 per group).


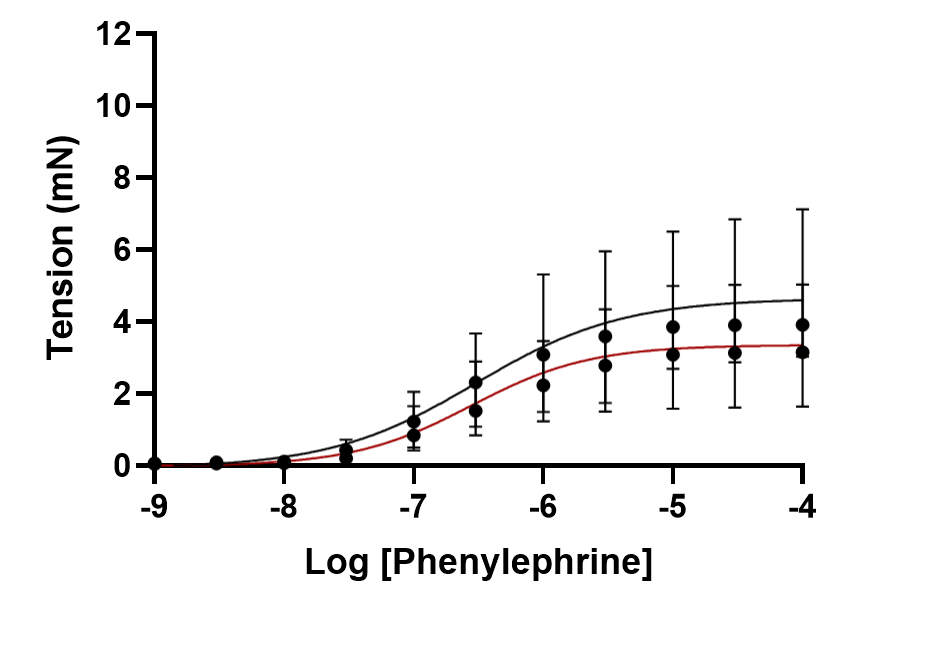
 **A B**

Thoracic Aortas

Mesenteric arteries


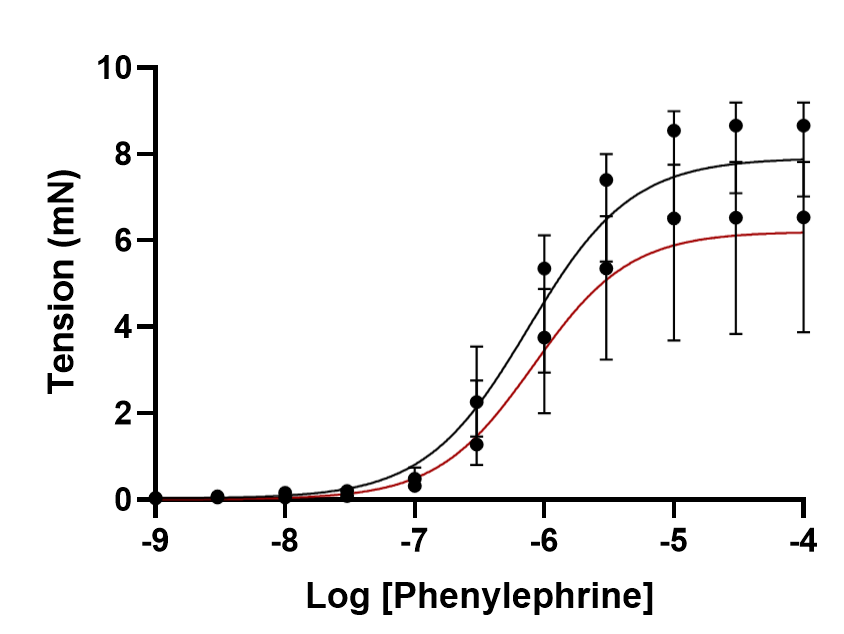


n = 8

n = 8

n = 8

Thoracic Aortas

Mesenteric arteries


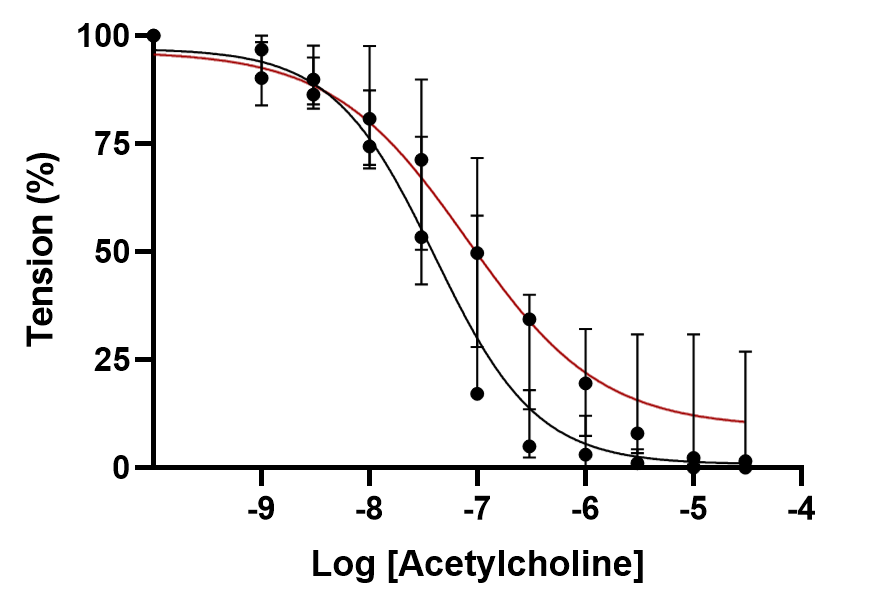

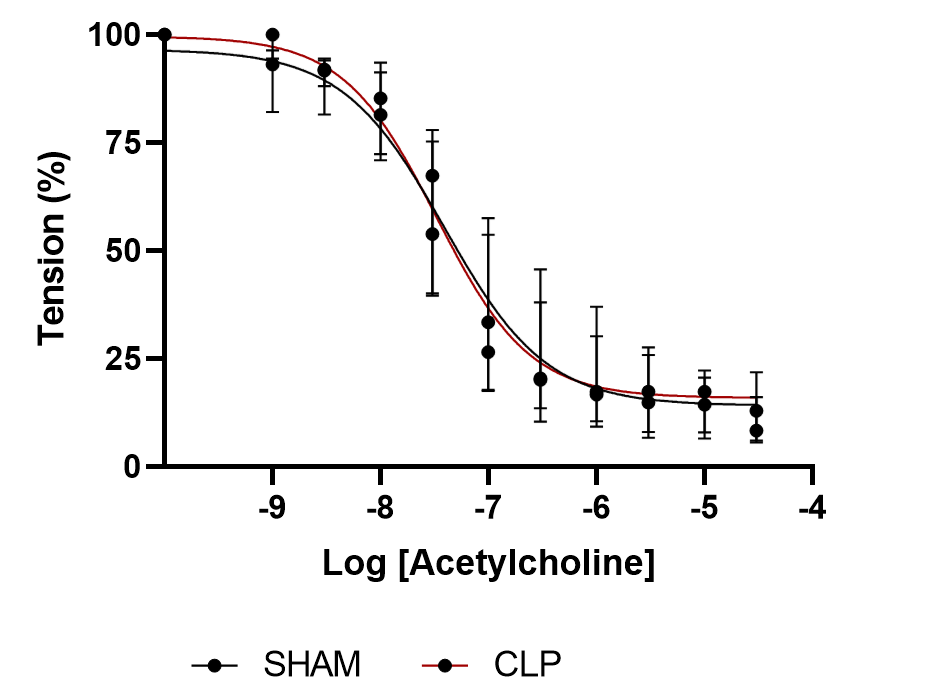
 **C D**

n = 8

n = 8

## **Figure S4: Analysis of NOS isoforms in heart tissue in SHAM and septic shock mice.**

(A) and (B) Relative protein expression of phosphorylated eNOS/eNOS and inductible NO synthase (iNOS) using Western blot. (C) RT-PCR relative quantification of RNA relative expression of neuronal NO synthase (nNOS) in the heart. The bracket indicates the corresponding blot. Data are expressed with median [25^th^; 75 ^th^] (n = 8 per group).


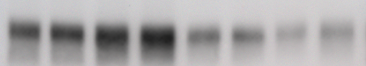

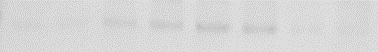
 **A B**  **C**

iNOS

peNOS


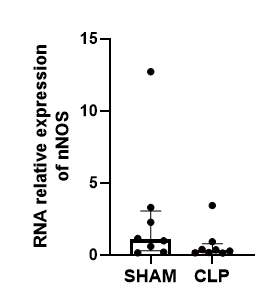

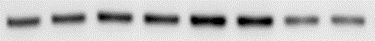
**
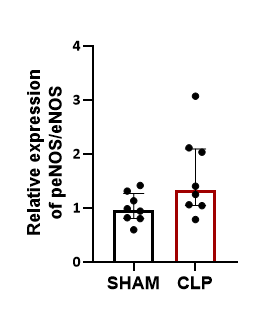
**

eNOS


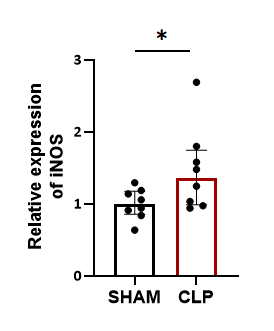


## **Figure S5: Analysis of inflammatory biomarkers in heart tissue in SHAM and septic shock mice without or with β3-AR modulation.**

(A), (B), (C), (D), (E) and (F) RNA relative expression of Endothelial selectin (E-selectin), Vascular Cell Adhesion protein 1 (VCAM-1), InterCellular Adhesion molecule protein 1 (ICAM-1) using RT-PCR. RPS29 was used as the reference gene for the analysis (n = 8 per group). (G) and (H) Relative protein expression of phosphorylated NFκB/NFκB using Western blot technique (n = 8 per group). The bracket indicates the corresponding blot. All data are expressed with median [25^th^; 75 ^th^].

**
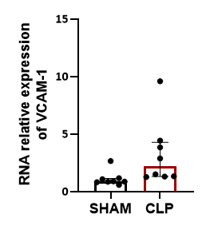

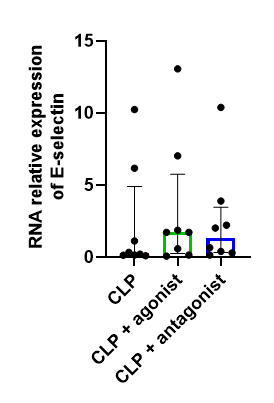

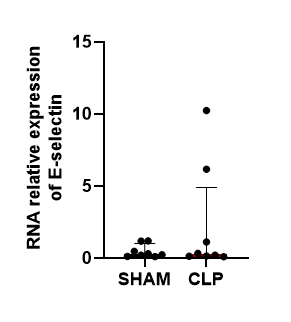
A B C**

**
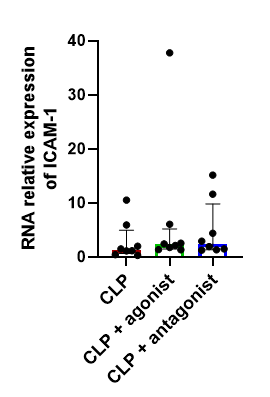

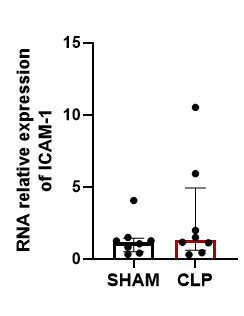

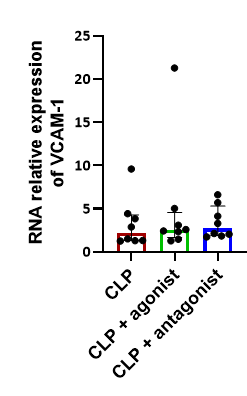
D E F**


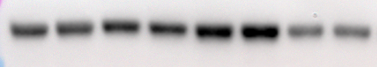

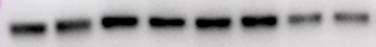

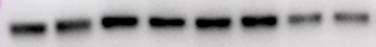

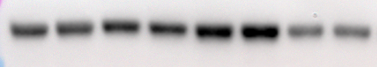
**G H**

NFκB

pNFκB

NFκB

pNFκB

pNFκB

NFκB


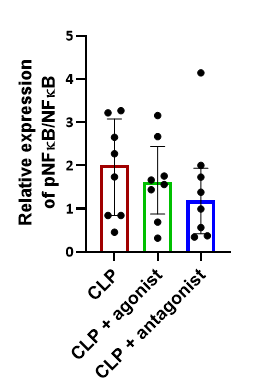
**
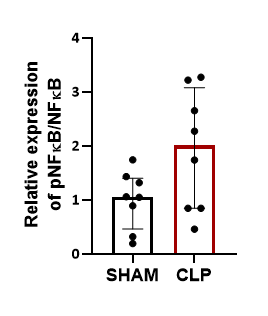
**

## **Figure S6: Evaluation of the vascular permeability in septic shock mice.**

(A) and (B) Lung wet/dry weight ratio. The weight of the dried lungs was subtracted from the freshly collected lungs and then normalized to the weight of the animal (n = 8 per group). (C) and (D) RNA relative expression of vascular endothelial cadherin (VE-cadherin) in the heart using RT-PCR. RPS29 was used as the reference gene for the analysis (n = 8 per group). Data are expressed with median [25^th^**;**75 ^th^].


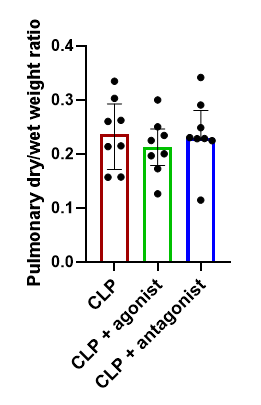

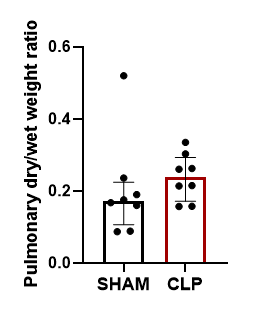
**A B**

**
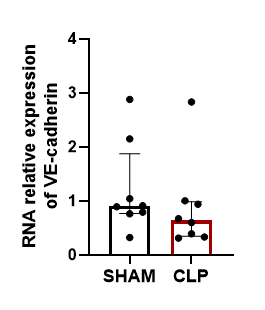
C D**


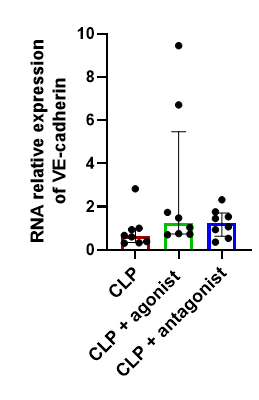


## **Figure S7: Variation in clinical score and weight of septic shock mice over five days.**

(A) The clinical score was calculated for each animal from day one to five. (B) Weight variation was monitored daily relative to the animal's pre-surgery weight. Statistical analysis was performed using mixed linear models. Data are expressed with median [25^th^;75 ^th^].

**
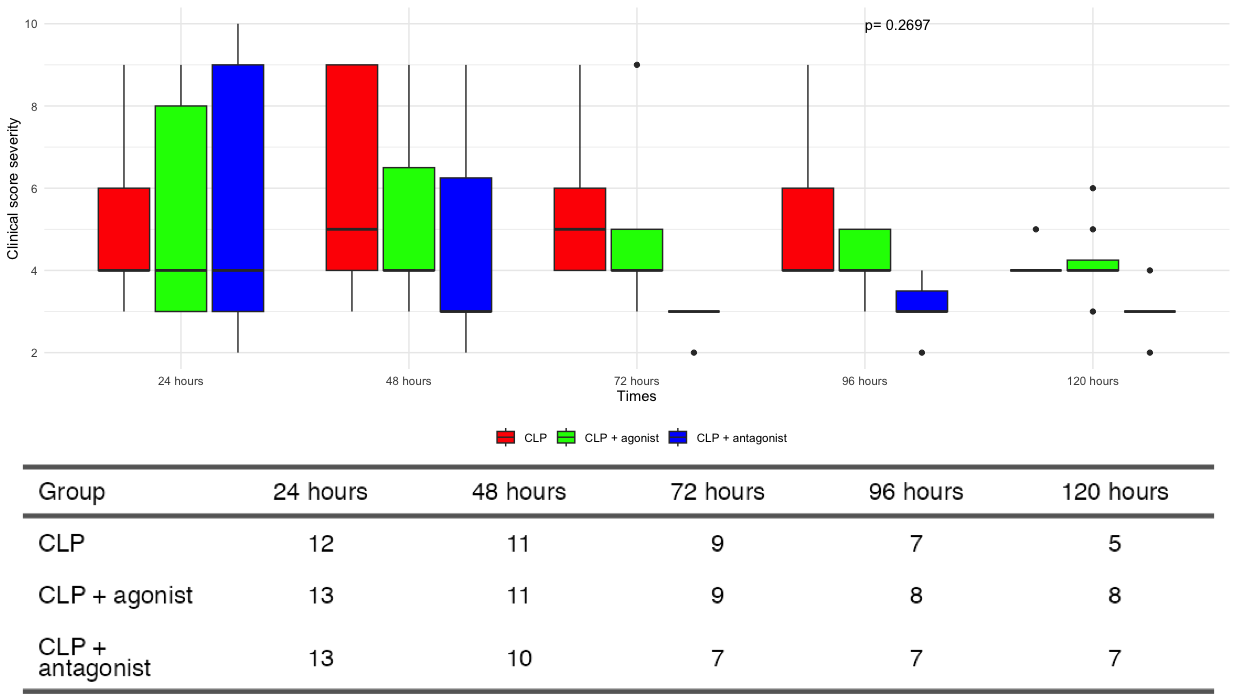
A**

**
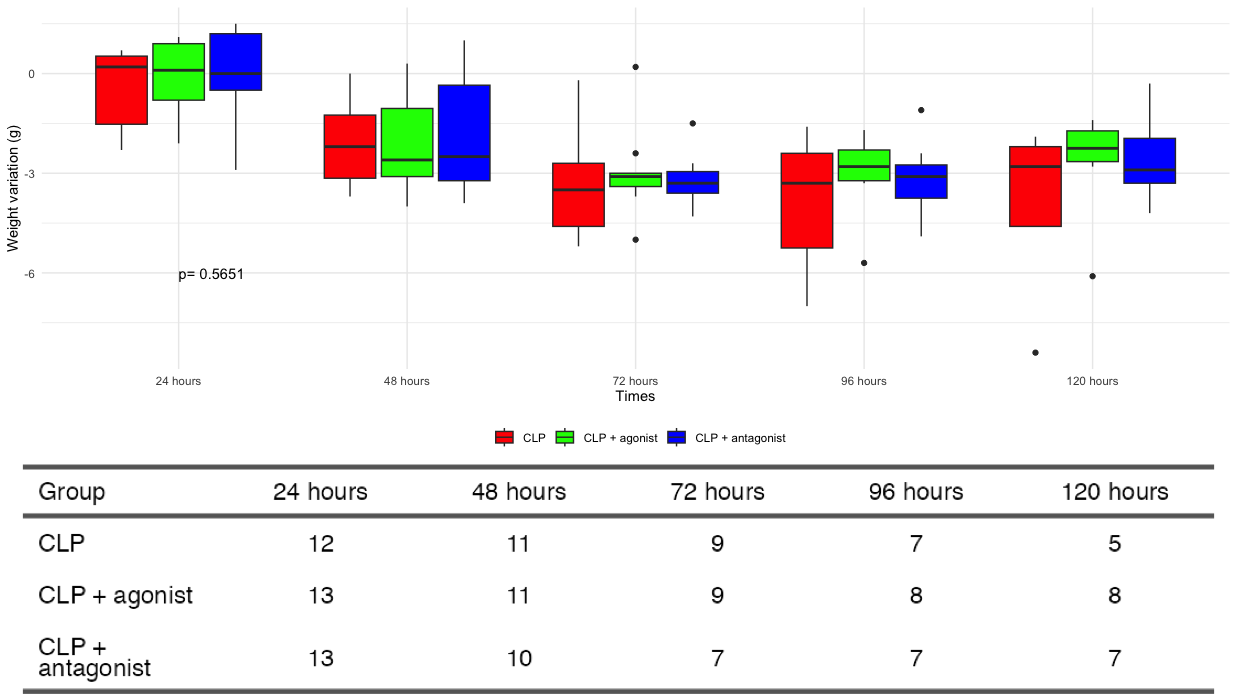
B**
